# Recurrent Posterior Reversible Encephalopathy Syndrome in an Adolescent Boy with End-Stage Renal Disease

**DOI:** 10.1155/2021/6675454

**Published:** 2021-02-16

**Authors:** Andrew Shieh, Natalie Darro

**Affiliations:** ^1^University of California, Irvine Medical Center, Irvine, CA, USA; ^2^Miller Children's & Women's Hospital Long Beach, Long Beach, CA, USA; ^3^Pediatrics Comprehensive Care, University of Utah Health, Salt Lake City, UT, USA

## Abstract

Posterior reversible encephalopathy syndrome (PRES), also known as reversible posterior leukoencephalopathy syndrome, is a neurological entity characterized by acute change in consciousness, visual impairment, headache, and seizures. It is associated with autoimmune disease, immunosuppressive agents, organ transplantation, acute glomerulonephritis, and sepsis. Typically, vasogenic edema is seen in the white matter of parieto-occipital lobes but can also involve atypical locations such as frontal lobes, thalamus, basal ganglia, and gray matter. While occurring extensively in adults, few cases, especially recurrent episodes, have been described in children. We report a case of recurrent PRES in a 17-year-old boy with end-stage renal disease on a peritoneal dialysis program who initially presented with hypertension and seizures. He emergently received intravenous antihypertensive medication with immediate and sustained improvement in his mental status. Information about recurrent PRES in children is limited because it is not commonly seen. We examine the clinical features of PRES and highlight important points for the diagnosis and management of this rare syndrome. This report demonstrates the importance of pediatricians to consider PRES in the differential diagnosis in children presenting with acute altered mental status. Blood pressure measurements, which are often overlooked in pediatric care, may assist in correctly diagnosing patients.

## 1. Introduction

Posterior reversible encephalopathy syndrome is a clinical-radiological condition with various etiologies that was first described in 1996 by Hinchey and his colleagues [1]. It is clinically characterized by altered mental status, seizures, headache, and visual disturbances. Classical neuroimaging features of PRES include cerebral vasogenic edema (leukoencephalopathy) without infarction predominantly in the parieto-occipital white matter of the brain [1, 2]. Although PRES is often reversible and clinical resolution is often achieved, it can lead to severe neurologic complications if not promptly recognized and treated [3]. To date, few studies of PRES in children with renal disease have been published, and as a result, expertise in the peculiar field of PRES remains lacking. The precise incidence of PRES or recurrence of PRES in children is not known and is thought to be rare. We present a case of recurrent PRES in an adolescent boy with end-stage renal disease on a peritoneal dialysis program to discuss the identification, diagnosis, and management of PRES in the pediatric population.

## 2. Case Presentation

Two days before visiting our hospital, a 17-year-old boy with end-stage renal disease had intermittent headaches and nausea. However, given improvement with acetaminophen, his parents did not believe he was ill. Soon after, he experienced consciousness disturbance and presented to the emergency department with a whole-body tonic-clonic seizure for fifteen minutes. The patient had no prior history of seizures.

He was initially diagnosed with anemia and chronic renal failure due to bilateral renal hypoplasia at 15 years of age. His glomerular filtration rate at the time of diagnosis was estimated to be 2 ml/min per 1.73 m^2^ using the Modification of Diet in Renal Disease Study equation. He had been on nightly peritoneal dialysis since his diagnosis. He was instructed to limit his daily intake of fluid to two liters, sodium to two grams, and phosphorus to one gram. Prior to arrival, he was receiving extended-release nifedipine, clonidine patch, furosemide, calcitriol, ergocalciferol, sevelamer carbonate, calcium carbonate, iron, and erythropoietin therapy. Further investigation with his parents revealed that the patient was not compliant with his medications or his daily fluid restriction.

On clinical examination, his vital signs revealed a temperature of 36.7°C, heart rate of 77 beats/min, respiratory rate of 25 breaths/min, and blood pressure of 210/120 mmHg. He required oxygen therapy to achieve oxygen saturation of 95%. The physical examination revealed a patient who was somnolent and incoherent, although neurologic exam (cranial nerves, sensory, and motor strength) was without focal deficits. Fundoscopic examination was normal and revealed no sign of optic disc edema. He had a peritoneal dialysis catheter secured to his abdomen.

**Figure 1:**
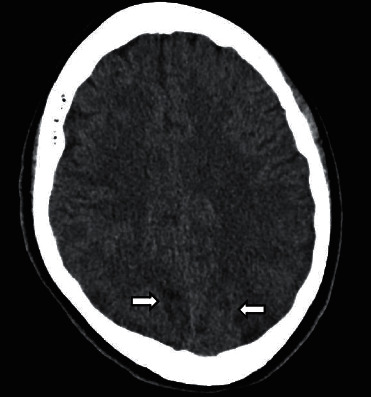
Axial computed tomography image revealing patchy hypodensity in the posterior parietal and occipital lobes, right greater than left. There are few tiny foci of hyperdense hemorrhage seen in areas of edema bilaterally.

In his laboratory findings, the white blood cell count was 11.5 k/uL, hemoglobin was 11.8 g/dL, blood urea nitrogen was 88 mg/dL, creatinine was 19.4 mg/dL, and glomerular filtration rate was estimated to be 3 ml/min per 1.73 m^2^. Urinary testing indicated 2+ protein, which was unchanged from his prior testing. There were no other remarkable laboratory parameters on blood and urinary testing. Cranial computed tomography revealed minimal scattered hemorrhage in areas of edema in bilateral parietal and occipital lobes (Figure 1). During his stay at the emergency department, he continued to have seizures and was treated with multiple doses of intravenous lorazepam. He was emergently intubated for airway protection. At this time, the presence of hypertension with disturbance of consciousness prompted us to consider the diagnosis of PRES. The differential diagnosis included acute neurological diseases such as ischemic stroke and cerebral venous thrombosis. Autoimmune encephalitis, central nervous system vasculitis, and unknown drug toxicity were also under consideration. Other differential diagnoses included meningitis and infectious encephalitis, particularly herpes simplex encephalitis, which would require rapid treatment with intravenous acyclovir. The patient was transferred to the pediatric intensive care unit and started on a continuous infusion of nicardipine.

**Figure 2:**
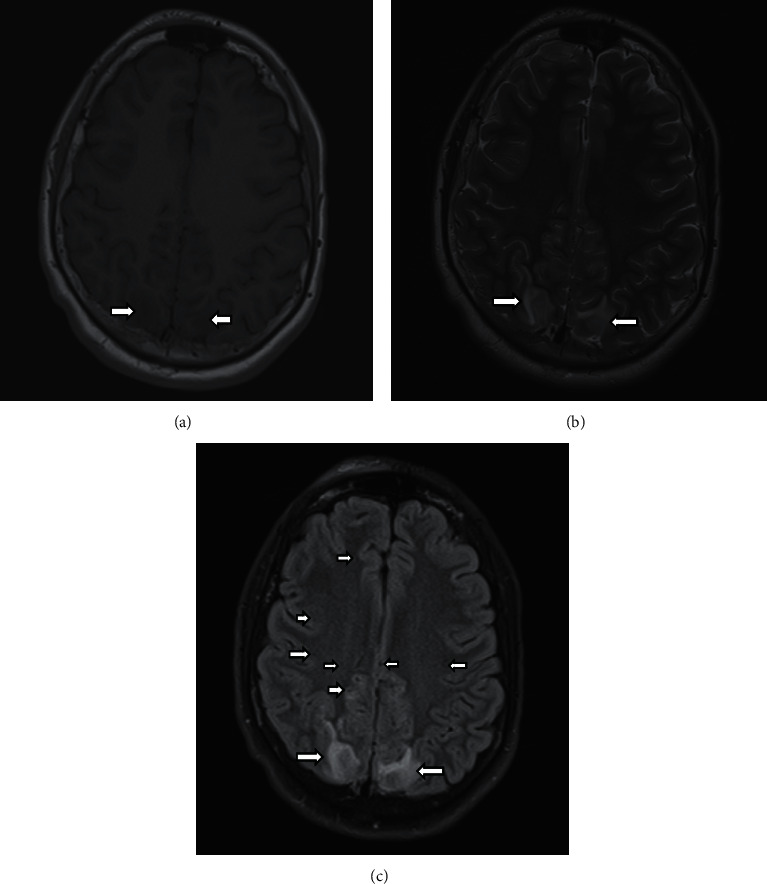
Axial T1-weighted (a), axial T2-weighted (b), and axial FLAIR (c) MRI images revealing increased signal consistent with edema in the cortex and subcortical white matter of the posterior parietal, temporal, and occipital lobes bilaterally. There are minimal scattered foci of hemorrhage in the anterior right anterior parietal lobe.

He underwent brain magnetic resonance imaging without contrast, which revealed low signal intensity on T1-weighted images and high signal intensities on T2-weighted and FLAIR images consistent with edema involving the cortical and subcortical regions of the posterior cerebral hemispheres (Figure 2). His echocardiogram revealed mild concentric left ventricular hypertrophy, normal left ventricular and right ventricular function, and no pericardial effusion. The patient was immediately treated with intravenous levetiracetam, continued on the infusion of nicardipine with careful monitoring of his blood pressure, and received his nightly peritoneal dialysis. The dose of nicardipine was carefully adjusted to reduce his systolic blood pressure by approximately 10% every hour. During the second day of hospitalization, the patient's consciousness improved gradually. He was extubated and had no seizure recurrence. His neurologic examination was normal. When his systolic blood pressure stabilized to 130 mmHg without the need for continuous infusion of nicardipine, he was restarted on his previous antihypertensive medications consisting of extended-release nifedipine, clonidine patch, and furosemide. His vital signs and electrolytes were carefully monitored for two days. The patient was educated on the importance of adherence to medications. The patient was discharged on the fourth day of hospitalization with a strict fluid restriction of two liters per day, and no adjustments were made to his medications.

One week later, the patient again presented to the emergency department with a headache and seizure lasting ten minutes. His blood pressure was high (180/110 mmHg) on arrival, and his physical examination including fundoscopy was normal. His blood urea nitrogen and creatinine concentrations were high, similar to his prior values. A repeat cranial computed tomography revealed no additional findings compared to his prior imaging. He did not require mechanical ventilation. As his findings were suggestive of recurrent PRES, he was transferred to the intensive care unit on a continuous infusion of nicardipine. He did not have any more seizures and his drowsiness improved overnight. The patient remained on the continuous infusion for one day, and after his blood pressure stabilized, he was monitored closely on furosemide, a higher dose of clonidine patch, and a higher dose of extended-release nifedipine for four days. He was continued on his nightly peritoneal dialysis. The patient was discharged on the fifth day of hospitalization with higher doses of his antihypertensive medications and no anticonvulsants.

## 3. Discussion

Our case describes an adolescent boy with end-stage renal disease (ESRD) on a peritoneal dialysis program due to bilateral renal hypoplasia diagnosed two years prior, who was hospitalized twice for PRES within one week. We briefly review the implications of hypertension in ESRD, address crucial principles in the diagnosis and management of PRES, and highlight the importance of frequent follow-up to monitor for recurrence of PRES in children.

Chronic kidney disease (CKD) and ESRD are catabolic states associated with progressive glomeruli and nephron destruction, which cause extracellular fluid overload and sodium retention. Renal scarring and regional ischemia cause increased response in the renin-angiotensin system and overactivity of the sympathetic system, which increases renin and angiotensin II release [4]. Angiotensin II is a vasoconstrictor that acts on the zona glomerulosa of the adrenal cortex to release aldosterone to increase water absorption and sodium uptake, alter baroreceptor reflexes, and stimulate the release of antidiuretic hormone from the pituitary gland to increase water uptake [5, 6]. There is also evidence that free radicals produced from oxidative stress cause increased cranial sympathetic outflow leading to excessive production of angiotensin II [5]. Subsequent hypervolemia and increased systemic vascular resistance result in hypertension which contributes to the progression of kidney disease.

Our patient was diagnosed with renal hypoplasia based on renal ultrasonography two years prior to his diagnosis of PRES. There is currently no consensus on the evaluation of hypertension in children discovered to have renal hypodysplasia. However, we recommend all children with persistent blood pressure above the 95^th^ percentile to be evaluated with a complete blood cell count, urinalysis, serum urea nitrogen level, serum creatinine level, electrolytes, and renal ultrasonography because other renal disorders may cause hypertension. If no other etiology is evident, serum renin level, serum aldosterone level, serum plasma steroid levels, thyroid studies, serum metanephrines, and urine metanephrines should be obtained [7]. Renal ultrasonography with Doppler and radionuclide renal scans can be used as screening tests for renovascular disease. Imaging studies with magnetic resonance angiography and renal vein renin measurements are necessary to evaluate renal vasculature. However, these tests are invasive and may not be available at all institutions due to the technical skill necessary.

Adequate volume control is crucial in the treatment of hypertension in children on dialysis, and antihypertensive medications should be considered if control of blood pressure remains poor. Salt-restricted diet (<2 grams/day) and fluid restriction (<2 liters/day) are often utilized. Loop diuretics are the diuretic class of choice in patients with ESRD. Angiotensin-converting enzyme inhibitors (ACEis) and angiotensin II receptor blockers (ARBs) are recommended because they decrease proteinuria and slow the progression of disease [7, 8]. However, the use of these medications is contraindicated in patients with bilateral renal artery stenosis, pregnancy, or persistent hyperkalemia. Consideration should also be given to the pharmacokinetic properties in regard to removal by dialysis. ACEis are mostly removed by hemodialysis with the exception of fosinopril, whereas ARBs and calcium channel blockers are not cleared by dialysis [9]. Our patient was initially treated with enalapril after his diagnosis but developed hyperkalemia unmitigated by diuretics. He was switched to a dihydropyridine calcium channel blocker (nifedipine) which helped to control his hypertension. Beta-blockers and centrally acting agents (clonidine) are also used as adjunctive agents to control blood pressure. Additionally, treatment of ESRD requires renal replacement therapy, specifically either dialysis or renal transplantation.

There is no consensus on recommended target blood pressure in children on dialysis. The ESCAPE trial in 2009 showed that intensified blood pressure management in children with CKD and hypertension receiving ramipril with target blood pressure less than the 50^th^ percentile led to slower progression of CKD compared to standard targets of 50^th^ to 90^th^ percentile [10]. As a result, the Kidney Disease Improving Global Outcomes guidelines recommended maintaining a systolic and diastolic blood pressure target less than the 50^th^ percentile in children with CKD stages II–IV [11]. We recommend a strict systolic blood pressure goal with a target at or below the 50^th^ percentile for gender, age, and height in children on dialysis to avoid complications.

Posterior reversible encephalopathy syndrome is a condition characterized by seizures, headache, nausea, vomiting, mental status changes, and visual disturbances associated with transient vasogenic edema seen on neuroimaging. Children with renal diseases and transplants are at the highest risk due to sudden increases in blood pressure. The estimated incidence of PRES in children with renal diseases is between 4% and 9%, although some patients may develop PRES without clinical seizures [2, 12, 13]. It is difficult to discuss PRES without discussing hypertensive encephalopathy, as both conditions are complications of malignant hypertension. Acute hypertensive encephalopathy is characterized by hypertension associated with neurological symptoms including headache, visual disturbances, seizures, focal deficits, and coma [14]. We recognize PRES as a subset of hypertensive encephalopathy with characteristic imaging features although, in practice, the two conditions lie in a continuum. It is crucial to recognize that patients with PRES may not have elevated blood pressure or vasogenic edema solely in the posterior lobes [15]. Patients with either diagnosis may develop microhemorrhages if there is no complete resolution of their radiological abnormalities [16]. Regardless of which term is used, recognition of the atypical radiological findings in PRES can reassure the clinician when a patient's clinical presentation is consistent with hypertensive encephalopathy. In both cases, antihypertensive treatment should commence urgently once the diagnosis is considered.

PRES is commonly seen in children who are on immunosuppressive therapy for hemato-oncological diseases such as leukemia, lymphoma, aplastic anemia, lymphohistiocytosis, autoimmune lymphoproliferative syndrome, and Evans syndrome [17, 18]. PRES is also commonly seen in children with kidney disease and renal transplantation [2, 13]. Other reported causes of PRES include immunosuppressive medications (cyclosporin A, tacrolimus) and cytotoxic chemotherapeutic agents. These agents have direct toxic effects on vascular endothelium in the brain, causing the release of vasoactive agents leading to edema [19–21]. There are also rare reports of PRES associated with hypertension seen in Henoch-Schönlein purpura and systemic lupus erythematosus [21, 22].

Vasogenic cerebral edema is the primary feature seen in PRES. Currently, there are three theories regarding the etiology of the edema. The first theory centers around the autoregulatory failure of cerebral blood flow due to severe hyperperfusion. When the mean arterial pressure acutely exceeds the upper limit of autoregulation, hyperperfusion leads to interstitial hydrostatic edema [2, 23, 24]. However, PRES may also occur in the absence of elevated arterial pressure, and the degree of cerebral edema does not directly correlate with the elevation of blood pressure [25, 26]. According to a second theory, hypertension activates the autoregulatory system which causes vasoconstriction of cerebral vessels. Vasospasm contributes to ischemia and cytotoxic edema in regions of limited arterial supply in the brain [12]. PRES may also result from endothelial dysfunction as seen in autoimmune diseases, end-stage renal disease, and transplantation [20, 21]. When blood pressure is elevated, vasoconstriction from autoregulation exacerbates endothelial dysfunction [26, 27].

There are currently no guidelines for the diagnosis of PRES. Seizures are the most common presentation, with the majority presenting as generalized tonic-clonic seizures. Other symptoms include altered mental status, lethargy, severe headache, nausea, vomiting, and vision impairment. Ophthalmological symptoms may include visual blurring, scotomas, hemianopsia, and cortical blindness [2]. Focal neurological deficits are uncommonly seen.

Magnetic resonance imaging (MRI) is considered the gold standard to diagnose PRES. Edema is classically seen in the white matter of the parieto-occipital regions bilaterally [1, 2]. T2-weighted images and fluid-attenuated inversion recovery (FLAIR) images frequently reveal hyperintensity in the posterior portions of the cerebral hemispheres. These findings are thought to be due to relatively few sympathetic innervations in the posterior circulation. Other patterns have also been described, including a holohemispheric pattern along the frontal, parietal, and occipital lobes in a watershed distribution. The superior frontal sulcus pattern describes predominant involvement in the frontal lobes. Partial manifestations of these patterns have also been described [20, 24]. Atypical sites of involvement include the cerebellum, basal ganglia, thalami, internal capsule, and brain stem. Cranial computed tomography (CT) can be quickly obtained to detect intracranial hemorrhage, but it has limited sensitivity for detecting PRES. Electroencephalography can show nonspecific slow waves and spikes and can be used to rule out subclinical status epilepticus but does not provide specific information regarding diagnosis. A cerebrospinal fluid examination should only be obtained if infectious or inflammatory disease is highly suspected.

In addition to vasogenic edema, the presence of hemorrhage and contrast enhancement on MRI has been described in literature [15, 16]. Previous larger studies report hemorrhage to occur in approximately 15%–17% of adult patients based on FLAIR, T2-weighted imaging, and nonenhanced CT [15, 16]. Susceptibility weighted imaging (SWI) is a newer sequence that has been shown to be more sensitive than traditional techniques in detecting cerebral microhemorrhages. In a study of 31 adult patients, microhemorrhage was detected in 58% of patients at presentation and remained in 65% of patients who received follow-up imaging within one year [28]. Preliminary studies reveal the use of SWI to depict microhemorrhages may improve prognostic outcomes, as the presence of PRES-related hemorrhage may potentially have poorer functional outcomes [29]. Further studies are required to elucidate the clinical significance of microhemorrhages on SWI in children.

Prompt recognition of PRES and initiation of therapy are important to prevent serious neurological sequelae. The management includes regulation of blood pressure, treatment of seizures, and elimination of causative medications. After cerebral hemorrhage and infarction are ruled out, blood pressure should be lowered by no more than 25% within the first hour of presentation, followed by a slower reduction afterward [30]. We recommend lowering the blood pressure more slowly to reduce the risk of secondary ischemia and possibly infarction. Intravenous antihypertensive medications including hydralazine, labetalol, and sodium nitroprusside are commonly used. Nicardipine infusion is very effective in lowering blood pressure, and the dose should be adjusted to achieve desired results. Electrolyte disturbances and fluid overload should be corrected [2]. Seizures may progress to status epilepticus and may require intravenous anticonvulsants for treatment. Lorazepam and diazepam are often first-line agents, while phenytoin and phenobarbital are commonly considered as second-line agents [2]. Midazolam can also be used. Discontinuation of calcineurin inhibitors should be considered, although this recommendation is controversial as withdrawal can cause graft rejection [2]. Some studies have reported good outcomes with dose reduction of immunosuppressive medication [19].

Most symptoms of PRES develop abruptly, and clinical resolution is typically achieved within a few days with prompt supportive management. The prevalence of PRES specifically in children with kidney disease is not established. Previous reports have documented PRES in children with ESRD and Henoch-Schönlein purpura [31, 32]. Limited cases of recurrent PRES have been published, and most recurrent cases describe adult patients with underlying diseases such as sickle cell disease, systemic lupus erythematosus, or leukemia with bone marrow transplantation who present with seizures [21]. The incidence of recurrence is estimated to be 4% in adult patients but remains unknown in pediatric patients [21]. In a cohort study of eighteen children with renal disease, two anephric patients each had three recurrent hypertensive episodes triggering PRES, with the earliest recurrence as early as five months after the initial episode [13]. These patients were found to have renal malignancy and steroid-resistant nephrotic syndrome. Recurrent PRES has been shown in these studies to be triggered by hypertension, infection or inflammation, the presence of organ dysfunction, and hemolysis secondary to endothelial injury [13, 21]. Our patient experienced a recurrence of PRES one week after discharge from the hospital which is much sooner compared to other cases previously described [13]. After symptomatic treatment of our patient during two hospitalizations, his clinical findings disappeared, and our patient has not had a recurrence of seizures.

There is debate whether PRES is truly reversible, as complications including intracranial hemorrhage and permanent neurologic sequelae have been reported [2, 3]. Unless symptoms do not completely resolve, a follow-up MRI is generally not necessary. However, we recommend frequent ambulatory blood pressure monitoring (ABPM) because it allows clinicians to monitor daytime blood pressure, sleep blood pressure, and mean blood pressure over 24 hours. The device is particularly useful to provide information that cannot be determined by office measurements, including white coat hypertension, determining the risk of organ damage, and response to pharmacologic therapy [33]. We emphasize it is necessary for children with CKD, especially ESRD, to be monitored closely with ABPM after treatment of PRES and every three months thereafter, as prolonged hypertension may result in neurological deficits and cerebral damage [2, 34].

The precise incidence of PRES in pediatric patients with kidney disease remains poorly established. Children with PRES and kidney disease are at higher risk for developmental delay, chronic imaging abnormalities with neurologic impairment, epilepsy, and recurrence of PRES [2, 13, 25]. Follow-up studies of children diagnosed with PRES are required to investigate the cognitive function of children before and after the diagnosis. We recommend long-term follow-up of all PRES patients with pediatric specialists to prevent recurrence. We hope further studies can elucidate the prevalence of PRES-related complications and the rate of recurrence in children.

## 4. Conclusion

Posterior reversible encephalopathy syndrome is rare in the pediatric population. The condition should be in the differential diagnosis for any patient with known malignancy, autoimmune disease, kidney disease, or organ transplantation who presents with hypertension, neurological symptoms, and disturbance of consciousness. Head MRI investigation often reveals edema in the subcortical white matter of the posterior portion of the cerebral hemispheres. Rigorous control of hypertension, seizures, and blood concentrations of calcineurin inhibitors are important strategies in managing PRES and preventing recurrence. We report a rare case of recurrent PRES in a child with end-stage renal disease on a peritoneal dialysis program. Further advances with MRI are required to understand the pathophysiology of this condition, improve diagnostic accuracy, and predict outcomes in patients with recurrent PRES. Further research is necessary to investigate factors considered to trigger the development of recurrent PRES in children.

## Data Availability

The clinical and laboratory data used to support the findings of this study are included within the article.
